# Familial cerebral cavernous malformations caused by a novel germline structural variant in the *KRIT1* gene

**DOI:** 10.1007/s10048-025-00847-2

**Published:** 2025-08-28

**Authors:** Robin A. Pilz, Matthias Begemann, Surema Pfister, Paranchai Boonsawat, Anita Rauch, Ingo Kurth, Ute Felbor, Matthias Rath

**Affiliations:** 1 Department of Human Genetics, University Medicine Greifswald and Interfaculty Institute of Genetics and Functional Genomics, University of Greifswald, Greifswald, Germany; 2 Center for Human Genetics and Genomic Medicine, Medical Faculty, RWTH Aachen University, Aachen, Germany; 3 Institute of Medical Genetics, University of Zurich, Zurich, Switzerland; 4 University Children’s Hospital Zurich, University of Zurich, Zurich, Switzerland; 5 Institute for Molecular Medicine, MSH Medical School Hamburg, Hamburg, Germany

**Keywords:** Cerebral cavernous malformation, Whole genome sequencing, Structural variant, Insertion

## Abstract

The detection of complex structural variants in patients with familial cerebral cavernous malformations (FCCM) remains challenging. Short-read whole genome sequencing was performed for a patient with strong clinical evidence of FCCM but negative results from previous genetic tests. The analysis revealed a large insertion of an intronic *KRIT1* fragment into a coding exon of *KRIT1*. This novel structural variant results in a frameshift and was classified as pathogenic. Predictive testing can now be offered to asymptomatic family members. This case expands the known mutation spectrum in FCCM and suggests that, after negative whole exome or gene panel sequencing, whole genome sequencing should be offered as a second-line diagnostic test.

## Introduction

Cerebral cavernous malformations (CCMs) are among the most common vascular anomalies of the central nervous system. Approximately one in 200 people has a CCM. The autosomal dominant form (familial CCM or FCCM) accounts for up to 7% of all cases and is caused by germline mutations in the *KRIT1* (also known as *CCM1*), *CCM2*, or *PDCD10* (aka *CCM3*) gene [1]. The probability of identifying a pathogenic variant is 87 to 98% for patients with a positive family history (FH). If the patient has a negative FH but multiple CCMs, the mutation detection rate is around 60% [1]. More than 400 different nonsense, frameshift, splice, and copy number variants in the *CCM* genes are listed as (likely) pathogenic in the ClinVar database [2]. While these mutations can be detected with high sensitivity in standard diagnostics, inversions, insertions, other complex structural variants (SVs), and deep intronic pathogenic variants remain a challenge [3–6]. However, detection of these variants is also extremely important for CCM families, as it is the only way to end the diagnostic odyssey and enable predictive analyses for family members. In the family described here, whole genome sequencing (WGS) identified the insertion of an intronic sequence into the coding region of the *KRIT1* gene, resulting in a frameshift and a premature stop codon.

## Results

### Clinical report

**Fig. 1:**
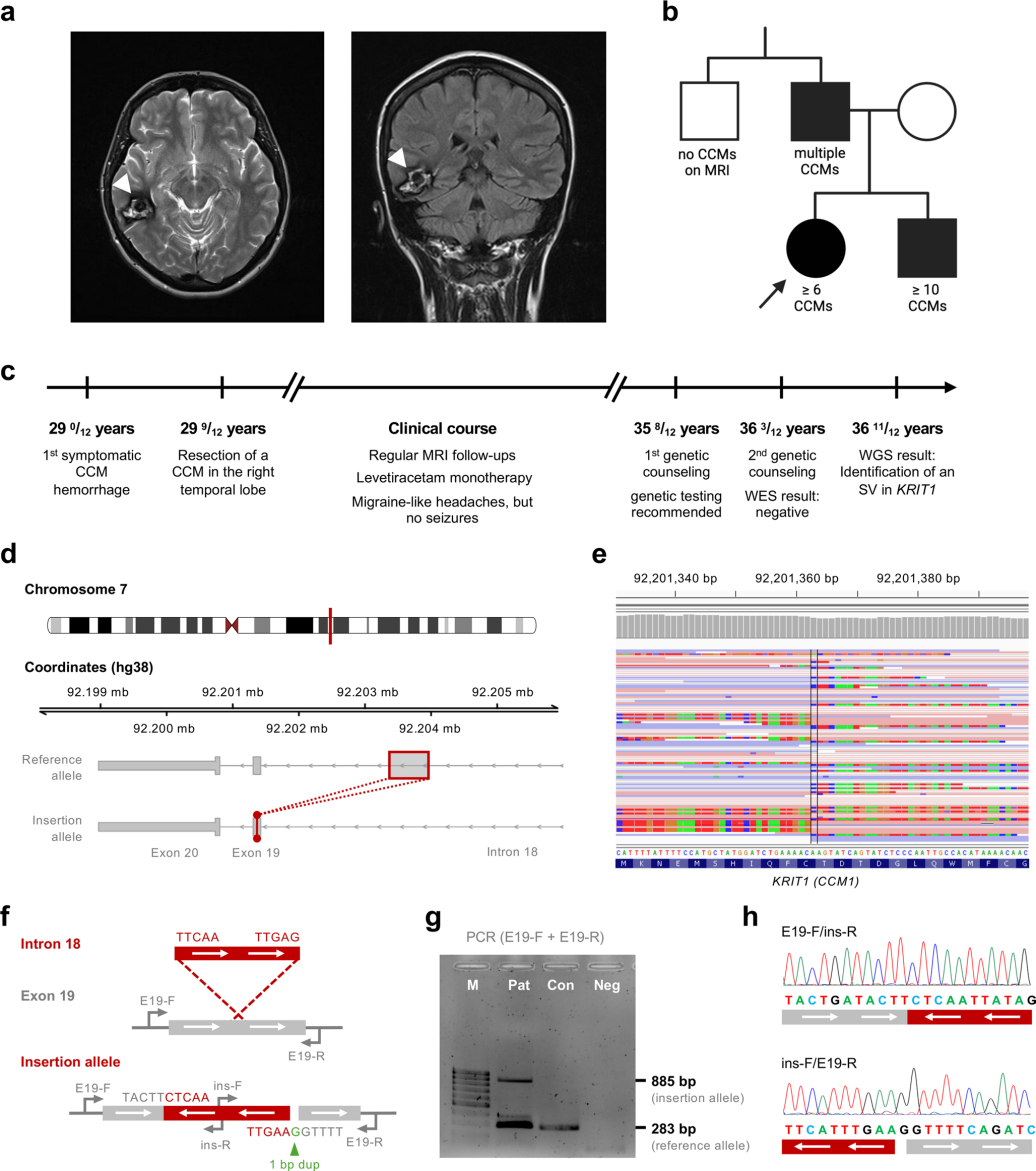
CCM family with a germline structural variant in the *KRIT1* gene. (**a**) Magnetic resonance imaging (MRI) showing a large CCM in the right temporal lobe (white arrowheads) of the patient. (**b**) Pedigree of the family with several CCM patients. The index case is marked with an arrow. (**c**) Clinical course of the patient. (**d**) Schematic illustration of the identified insertion of an intronic sequence into exon 19 of the *KRIT1* gene. The inserted sequence was found in an inverted orientation. (**e**) Alignment of the WGS reads. (**f**) Scheme of the confirmation approach with location of the PCR primers. (**g**, **h**) Confirmation of the insertion by PCR (**g**) and fine mapping of the breakpoints by Sanger sequencing (**h**). Sequencing also revealed the presence of an additional 1 bp (bp) duplication at the exonic breakpoint. Pat = index patient; Con = healthy control; Neg = negative control

The index patient had a first CCM hemorrhage at the age of 29 and later presented with focal impaired awareness seizures. Magnetic resonance imaging (MRI) revealed multiple CCMs in her cerebrum and brainstem (Fig. 1a). The family history was highly suggestive of FCCM (Fig. 1b). The patient’s brother and father both had seizures and multiple CCMs on MRI. Due to bleeding from two CCMs, the father had also experienced temporary hearing loss. After resection of a large CCM in her right temporal lobe and initiation of levetiracetam monotherapy, the index patient remained seizure free (Fig. 1c). At the age of 35, the patient was referred for genetic counseling to discuss the implications of the suspected hereditary basis of her CCMs.

### Genetic investigations

Genomic DNA for whole exome sequencing (WES) was extracted from a peripheral blood sample. The library was prepared and captured using the Twist Exome 2.0 kit (Twist Bioscience) and sequenced on a NovaSeq 6000 system (Illumina Inc., San Diego, CA, USA). 98% of target bases were covered with ≥ 20×. However, no pathogenic variant could be identified. Due to the strong clinical evidence of FCCM, we enrolled the patient in our CCM study and performed WGS using the DNA PCR-free kit (Illumina Inc.). Sequencing on a NovaSeq 6000 system resulted in a mean coverage of 41×. 92.5% of target bases were covered with a minimum of 20×. WGS data analysis was performed with the Illumina DRAGEN-Pipeline (Version: 07.021.645.4.0.3) using hg38 reference genome and the emedgene software (Illumina Inc.). In the bioinformatic analysis, a large insertion in the coding region of the *KRIT1* gene [RefSeq: NM_194456.1] was identified. However, this variant was only called by the SV-Caller (DRAGEN-MANTA4.2, calling_methodology: SV_SPLIT_END) of the emedgene bioinformatic tool set. The detection of SVs with the Manta caller is based on a scoring system which integrates various pieces of evidence and reflects the confidence in the calls. For each potential variant, a quality score is derived from combined paired and split-read evidence and local assembly, respectively [7]. Unsurprisingly, the GATK variant callers failed to identify the insertion. An inspection of the WGS data in the Integrative Genomics Viewer (IGV) revealed many split reads that mapped to exon 19 of the *KRIT1* gene, but also to a sequence approximately 1.7 kb away in intron 18 of *KRIT1* (Fig. 1d, e). PCR amplification and Sanger sequencing confirmed the insertion of a 601 bp fragment from intron 18, which was inserted in an inverted orientation into exon 19. In addition, a 1 bp duplication was identified at the exonic breakpoint (Fig. 1f-h). As the breakpoints of the inversion occurred in an exon, split reads were also retrospectively observed in the exome sequencing data. Following the HGVS recommendations, the variant was described as: LRG_650t1:c.2086_2087ins[2026-2243_2026-1643inv;G]. This SV presumably results in a frameshift with a premature stop codon [LRG_650p1:p.(Cys696Serfs*4)]. The variant is localized in the penultimate exon of *KRIT1*. However, since several loss-of-function variants downstream of the SV are listed as (likely) pathogenic in the ClinVar database and the variant most likely triggers nonsense-mediated mRNA decay, the insertion was classified as pathogenic (ACMG criteria: PVS1 + PM2 + PP4) [8, 9]. Symptomatic and asymptomatic relatives of the index patient were offered diagnostic and predictive analyses, respectively.

## Discussion

While genetic counseling of patients with confirmed FCCM is straightforward [10], cases with negative test results still pose a dilemma for genetic counseling and clinical management. Since no reliable statement about the risk of recurrence can be made and no predictive analyses can be offered, affected individuals remain in a state of uncertainty. The identification of a large insertion in *KRIT1* highlights the role of SVs in FCCM and provides further evidence to recommend WGS as a second-line diagnostic test for mutation-negative patients. Since up to 98% of CCM cases with a positive FH have simple single nucleotide, indel or copy number variants [11], second-line WGS is rarely necessary in this group, but very effective. In our CCM cohort, there is only one unsolved case with a suspicious but not entirely conclusive FH [1].

In addition to the case described here, three other CCM patients with SVs have been described [3–5]. Two of these also had a positive FH (Table 1). However, the current predominance of familial cases is most likely due to selection bias. Since the negative predictive value of genetic testing is significantly lower in patients with a positive FH, these cases are more likely to undergo additional WGS in a research setting. Considering that one of the three previously described patients with an SV was a sporadic case and that the median genome-wide de novo mutation rate for SVs has been reported to be 0.29 per generation [12], SVs are also likely to be a relevant but probably underestimated mutation type in CCM cases with negative FH.

**Table 1:** Germline structural variants (SVs) in the *CCM* genes

Case	I	II	III	IV
**Reference**	Spiegler et al. 2018 [3]	Pilz et al. 2020 [4]	Chaussenot et al. 2024 [5]	This study
**Personal history of the index case**	two symptomatic CCMs	multiple symptomatic CCMs	multiple symptomatic CCMs	multiple symptomatic CCMs
**Family history for CCM**	positive	negative	positive	positive
**Type of mutation**	inversion (24 kb)	insertion (294 kb)	complex chromosomal rearrangement (267 kb)	insertion (0.6 kb)
**Affected gene**	*CCM2*	*CCM2*	*CCM2*	*KRIT1*
**Sanger sequencing of** ***KRIT1***, ***CCM2***, **and** ***PDCD10***	no pathogenic variant	*- not performed -*	no pathogenic variant	*- not performed -*
**Gene panel sequencing**	*- not performed -*	**insertion identified**	*- not performed -*	*- not performed -*
**NGS-based CNV analysis/MLPA/QMPSF for** ***KRIT1***, ***CCM2***, **and** ***PDCD10***	no pathogenic variant	no pathogenic variant	no pathogenic variant	no pathogenic variant
**WES**	no pathogenic variant	*- not performed -*	*- not performed -*	no pathogenic variant
**WGS**	**inversion identified**	*- not performed -*	**complex chromosomal rearrangement identified**	**insertion identified**
**Additional genetic analysis**	amplicon-based sequencing of the entire genomic regions of *KRIT1*, *CCM2*, and *PDCD10* (negative for the index case)	**FISH analysis confirmed the unbalanced insertion that originated from 1p12-p11.2**	**sequencing of the cDNA (negative for the index case**,** but loss of heterozygosity in** ***CCM2*** **cDNA of her mother)**	**targeted manual re-analysis of WES data confirmed the findings of the WGS analysis**

Listed are variants described in the literature or identified in this study. Unbalanced gains or losses of coding DNA [= copy number variations (CNV)], which can be detected by standard diagnostics, are not included

## Conclusion

Due to their high diagnostic yield and good cost efficiency, WES or gene panel sequencing will remain the first-line test for CCM patients. However, apparently mutation-negative patients with strong clinical evidence of FCCM should be offered second-line WGS to also identify complex SVs or deep intronic pathogenic variants.

## Data Availability

The NGS data that support the findings of this study are not publicly available due to reasons of sensitivity and data protection regulations concerning personal data. They are available from the corresponding author upon reasonable request.

## Abbreviations

ACMG: American College of Medical Genetics and Genomics
CCM: Cerebral Cavernous Malformation
*CCM2*: Cerebral Cavernous Malformation 2
CNV: ​Copy Number Variation
FCCM: Familial Cerebral Cavernous Malformation
FERM: F for protein 4.1, E for ezrin, R for radixin, M for moesin
FH: Family History
FISH: Fluorescence In Situ Hybridization
HGVS: Human Genome Variation Society
IGV: Integrative Genomics Viewer
*KRIT1*: Krev Interaction Trapped 1
MLPA: Multiplex Ligation-dependent Probe Amplification
MRI: Magnetic Resonance Imaging
NGS: Next-generation Sequencing
PCR: Polymerase Chain Reaction
*PDCD10*: Programmed Cell Death 10
QMPSF: Quantitative Multiplex PCR of Short Fluorescent Fragments
SV: Structural Variant
WES: Whole Exome Sequencing
WGS: Whole Genome Sequencing
